# Utilising speech-derived biomarkers to detect Alzheimer's disease with BERT-based language models: a machine learning study

**DOI:** 10.3389/frai.2026.1875702

**Published:** 2026-08-31

**Authors:** Zara Khanna, Dean Ho, Alexandria Remus, Marlena Raczkowska, Railey Montalan, Pramit Saha

**Affiliations:** 1 The Institute for Digital Medicine (WisDM), Yong Loo Lin School of Medicine, National University of Singapore, Singapore, Singapore; 2 The N.1 Institute for Health, National University of Singapore, Singapore, Singapore; 3 Department of Biomedical Engineering, College of Design and Engineering, National University of Singapore, Singapore, Singapore; 4 Department of Pharmacology, Yong Loo Lin School of Medicine, National University of Singapore, Singapore, Singapore; 5 The Bia-Echo Asia Centre for Reproductive Longevity and Equality, Yong Loo Lin School of Medicine, National University of Singapore, Singapore, Singapore; 6 Singapore's Health District @ Queenstown, Yong Loo Lin School of Medicine, National University of Singapore, Singapore, Singapore; 7 Heat Resilience & Performance Centre, Yong Loo Lin School of Medicine, National University of Singapore, Singapore, Singapore; 8 Human Potential Translational Research Programme, Yong Loo Lin School of Medicine, National University of Singapore, Singapore, Singapore; 9 Office of the Deputy President (Research and Technology), National University of Singapore, Singapore, Singapore; 10 Department of Engineering Science, University of Oxford, Oxford, United Kingdom

**Keywords:** Alzheimer's disease, artificial intelligence, BERT, dementia detection, machine learning, natural language processing, speech biomarkers, transformer models

## Abstract

**Background:**

Early diagnosis is critical for effective management of Alzheimer's disease (AD). While prior studies have shown that speech features can be indicative of AD, most existing work consolidates multiple biomarkers, making it difficult to isolate the contribution of individual features.

**Objective:**

This study systematically isolates individual speech biomarkers to quantify their distinct contributions to AD classification performance of language models (LMs) and determine whether targeted biomarker selection improves over consolidated feature sets.

**Methods:**

We processed speech transcriptions from DementiaBank to surface discriminatory speech biomarkers—verbal pauses, disfluencies, and unintelligible words. We then fine-tuned and evaluated lightweight LMs (BERT, AlBERT, and DistilBERT) on these biomarker-conditioned transcripts for automatic AD classification.

**Results:**

Pauses emerged as the most discriminatory speech biomarker (F1 = 0.8326), significantly outperforming the No-biomarker baseline and other biomarkers. Combining all biomarkers degraded performance relative to pauses alone on average across models, though the effect was model-dependent: BERT's All-biomarkers condition exceeded its own No-biomarker baseline, suggesting that feature combination benefits higher-capacity models. BERT yielded the best performance (F1 = 0.8154) across conditions.

**Conclusions:**

Selective use of speech biomarkers such as pauses can meaningfully improve AD detection with lightweight LMs, suggesting that targeted biomarker selection may offer a more interpretable and clinically actionable path than broad feature consolidation.

## 1 Introduction

Alzheimer's disease (AD) is a progressive neurodegenerative disease and is the most common cause of dementia. AD develops through the buildup of amyloid plaques in the brain, leading to cognitive decline, with severe memory loss as a primary symptom (Larson et al., 1992). There is currently no cure for AD, but progression to later-stage dementia can be slowed by receiving appropriate treatment in earlier stages of the disease (Fekete et al., 2022). For this reason, early diagnosis is critical for effective management.

Currently, AD diagnosis relies on a combination of clinical assessment, neuropsychological evaluation, and neuroimaging. Clinical screening tools, such as the Cookie-Theft picture description task, are commonly used by trained clinicians to elicit spontaneous speech for analysis of markers of linguistic and cognitive decline (Lin et al., 2013). Recent research has shown that AI approaches, particularly language model (LM)-based methods, can be used to automatically detect AD from speech-derived prosodic and lexical features without the reliance on trained clinicians for initial screening (Luz et al., 2018; Ross and Monnot, 2011; Ahmed et al., 2013). Despite this progress, a key gap remains: most existing studies consolidate multiple biomarkers together, leaving individual feature contributions unexplored (Lopez-de Ipiña et al., 2015; Mirheidari et al., 2017). Which specific biomarkers are most discriminatory remains an open question. The answer determines whether diagnostic tools should combine features or target a single informative signal.

Key speech biomarkers associated with AD include prosodic features such as extended pauses and disfluencies, as well as lexical markers such as unintelligible words and vocabulary errors (Strimbu and Tavel, 2010; Ramanarayanan et al., 2022; Fritsch et al., 2019). These features reflect the underlying cognitive and linguistic decline characteristic of AD and are detectable from spontaneous speech transcripts. Table 1 summarises the key speech biomarkers discussed in this study. Weiner et al. (2017) documented a word-error rate of 0.58 in dementia patients. Models trained on semantic speech features detect dementia at 0.82 accuracy (Fraser et al., 2015). Prior work recommends directly comparing individual biomarkers to identify which drive AD detection most effectively (Petti et al., 2020).

**Table 1:** Key speech biomarkers associated with Alzheimer's disease.

Biomarker	Linguistic manifestation	Neurological basis
Disfluencies	Filled hesitation sounds (e.g., *um, uh*), false starts, and phonological fragments	Frontal-executive word-retrieval failure and disrupted phonological encoding; frequency is elevated in AD relative to age-matched controls (Strimbu and Tavel, 2010; Ramanarayanan et al., 2022)
Extended pauses	Silent intervals exceeding 1.5 s within propositional speech	Slowed lexical access and impaired semantic processing; long-pause ratio correlates inversely with hippocampal volume and increases with dementia severity (Khodabakhsh et al., 2015)
Unintelligible words	Segments phonetically unclear or lexically unrecoverable at transcription	Degradation of phonological and semantic representations; prevalence increases with disease progression (Fritsch et al., 2019)

Summary of the three biomarker types, their linguistic manifestation in spontaneous speech, and their established neurological basis (Strimbu and Tavel, 2010; Ramanarayanan et al., 2022; Fritsch et al., 2019; Khodabakhsh et al., 2015).

Normal ageing produces some word-finding difficulty and occasional disfluency. But pause events are substantially more frequent and severe in AD: individuals with AD show pauses approximately 1.2 standard deviations longer than age-matched controls, compared to 0.62 standard deviations for mild cognitive impairment (Khodabakhsh et al., 2015). Pauses exceeding 2 s are nearly absent in healthy older adults and increase systematically with dementia severity. They also correlate inversely with hippocampal volume, suggesting an anatomical basis for the biomarker signal rather than a purely age-related effect.

LMs are pre-trained on large text corpora for a variety of natural language processing (NLP) tasks (Zhou C. et al., 2025). The most prevalent architectures involve Transformers (Vaswani et al., 2017), which produce contextual representations well-suited to downstream classification tasks. Bidirectional Encoder Representations from Transformers (BERT) (Devlin et al., 2019) is among the most widely adopted, and Guo et al. reported BERT achieving 0.82 accuracy for dementia detection (Guo et al., 2021). Yuan et al. applied fine-tuned BERT to disfluencies (Yuan et al., 2020a) and to pauses (Yuan et al., 2020b) separately for AD detection. But these studies used different protocols, corpora, and evaluation settings. No existing work compares individual biomarkers under a controlled framework, so their relative discriminatory power remains unknown. More recent approaches include deep ensemble learning with transformer models (Latif et al., 2025; Chakravarti and Shivakanth, 2025), NLP-based classification from connected speech (Balabin et al., 2025), and the adoption of large language models (LLMs) such as Llama and multimodal architectures (Dubey et al., 2024; Kamath et al., 2025; Ding et al., 2024).

We systematically compare three individual speech biomarkers—pauses, disfluencies, and unintelligible words—alongside their combination and a No-biomarker baseline, holding model architecture, corpus, and evaluation protocol fixed across all conditions. Using speech transcriptions from the DementiaBank Pitt corpus, we fine-tuned three BERT-based LMs (BERT, AlBERT, and DistilBERT) on five distinct biomarker conditions and evaluated their performance using standard classification metrics. This directly addresses the recommendation to compare and isolate biomarker contributions (Petti et al., 2020), and informs the design of more targeted, interpretable clinical screening tools for AD. The study contributes: (1) a systematic isolation of three speech biomarkers under a fixed model, corpus, and evaluation protocol, enabling direct comparison; (2) the finding that feature combination degrades rather than improves average performance, with model-dependent exceptions; and (3) a model-architecture comparison under controlled biomarker conditions.

## 2 Materials and methods

### 2.1 Overview

**Figure 1:**
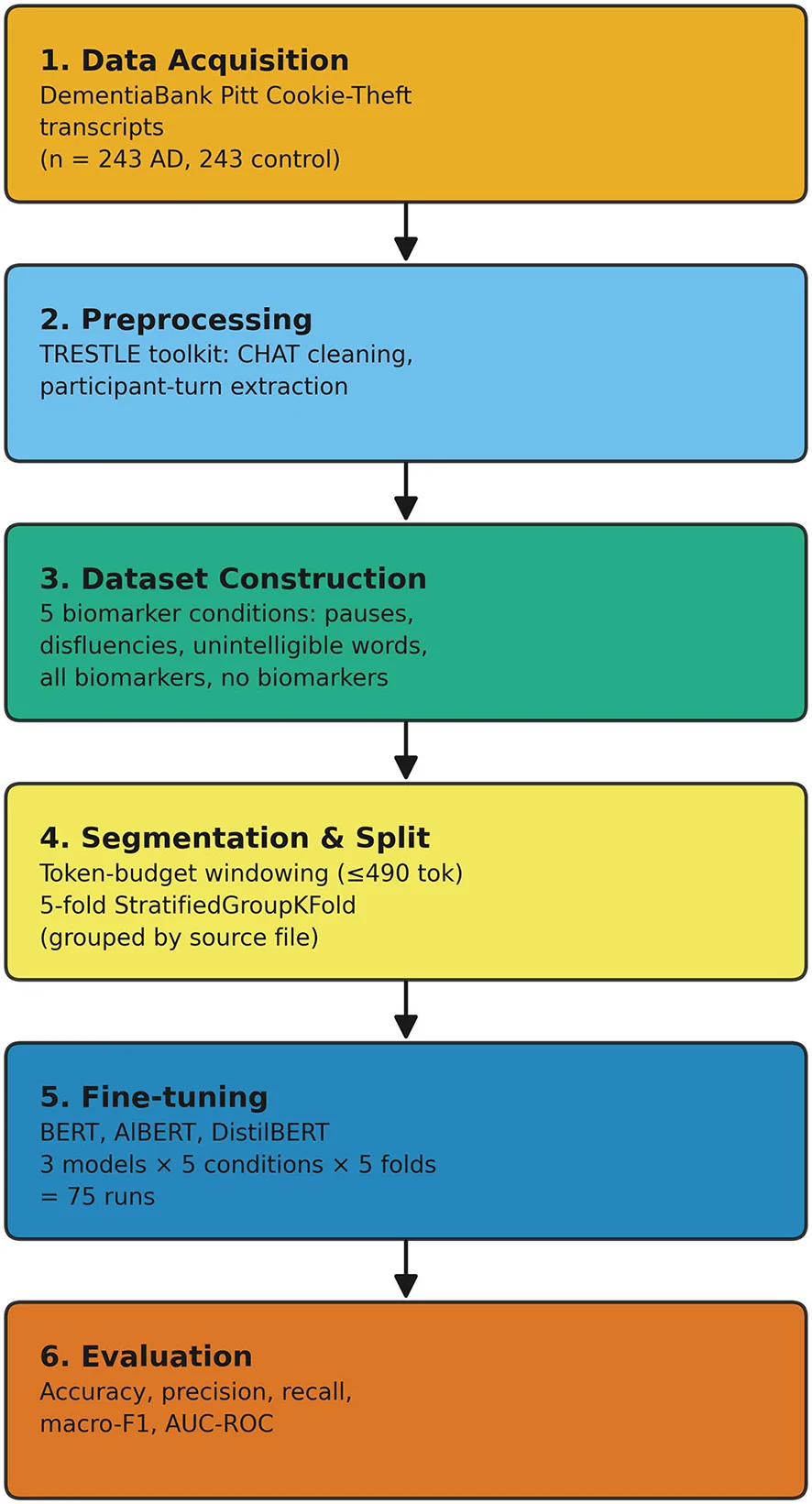
Study workflow overview. The six-step pipeline: (1) data acquisition from DementiaBank Pitt corpus; (2) preprocessing with the TRESTLE toolkit; (3) dataset construction with five biomarker conditions; (4) segmentation and five-fold stratified cross-validation split; (5) fine-tuning of BERT, AlBERT, and DistilBERT; and (6) model evaluation.

We utilised a six-step pipeline (Figure 1): (1) data acquisition from the DementiaBank Pitt corpus; (2) preprocessing with the TRESTLE toolkit; (3) dataset construction by isolating individual speech biomarkers into five distinct experimental datasets; (4) token-budget windowing and five-fold stratified cross-validation; (5) fine-tuning of BERT, AlBERT, and DistilBERT on each dataset; and (6) evaluation with standard classification metrics.

### 2.2 Data acquisition

We used DementiaBank (Lanzi et al., 2023), a multimodal dataset of transcribed conversations between participants (patients with AD or healthy controls) and clinical investigators. It forms a subset of TalkBank, a broader infrastructure for studying how speech and communication are affected in dementia. Participants ranged in age from 44 to 91 years, with a mean age of 70 years. Participant demographics are summarised in Table 2.

**Table 2:** Participant demographics.

Group	*N*	Age (years)	Sex (M/F)	Education (years)
AD (probable)	222	72.0 ± 8.3	80/163	18.5 ± 5.1^a^
AD (possible)	21			
Control	243	64.9 ± 7.7	89 / 154	N/A

Age and education: mean ± SD. Education data are available for AD participants only; control participant education is not encoded in the Pitt corpus .cha transcript files. MMSE scores were unavailable in the transcript-level metadata files. Complete cohort demographics are in the DementiaBank characterisation study (Lanzi et al., 2023).^a^Education available for 223 of 243 AD participants; 20 files have missing metadata.

The Cookie-Theft recordings from the Pitt subset were used, collected by the University of Pittsburgh School of Medicine. The Pitt Cookie-Theft subset contains transcripts from participants spanning a range of diagnoses. For this study, we restricted the dataset to probable and possible AD diagnoses (the positive class) and healthy control participants (the negative class), and balanced the two groups to equal class sizes, yielding 243 AD transcriptions (222 probable AD, 21 possible AD) and 243 control transcriptions. The Pitt corpus contained 255 transcriptions meeting the probable or possible AD criterion; 243 were randomly sampled to match the number of available control transcriptions, which were all used. These transcriptions included accompanying annotations for prosodic features of speech, such as disfluencies, pauses, and unintelligible words. Balancing was performed to ensure equal class representation and to avoid classifier bias toward a majority class.

### 2.3 Preprocessing

The transcripts were pre-processed using the Toolkit for Reproducible Execution of Speech, Text and Language Experiments (TRESTLE) toolkit (Li et al., 2023). The TRESTLE toolkit offers preprocessing scripts for text and audio data from corpora like DementiaBank, which use the CHAT format and Praat's TextGrid annotations. CHAT and Praat are both formatting conventions for preprocessing text and audio data, respectively. In particular, the following preprocessing steps were applied:

- Other transcription annotations such as the patient clearing their throat, taking a breath, etc., were removed.
- Lexical markings such as parentheses, square brackets, slashes, etc., were removed.
- Text formatting such as capitalising sentences and splitting sentences into separate lines was applied.

The toolkit was also used to isolate specific biomarkers (the transcription annotations) and remove all other remaining biomarkers from the dataset. Table 3 shows the remaining speech biomarkers investigated in this study.

**Table 3:** CHAT annotation codes for speech biomarkers retained in this study.

Biomarker	CHAT code(s)	Example	Condition(s)
Disfluencies	&- prefix: filled pauses / filler words &+ prefix: phonological fragments, false starts	&-um, &+su	disfluencies, all_biomarkers
Extended pauses	(...) and longer (≥3 dots); proxy for silent intervals >1.5 s. Short/medium markers (.) and (..) excluded.	(...)	pauses, all_biomarkers
Unintelligible words	xxx: phonetically unclear segment yyy: intonation recoverable, lexical form unclear www: word intentionally not transcribed	xxx, yyy, www	unintelligible, all_biomarkers

Each biomarker type maps to specific CHAT transcript codes (Li et al., 2023). The *Condition* column indicates which experimental dataset(s) retain each biomarker type; the no_biomarkers condition removes all three types.

The CHAT annotation codes used for each biomarker type were as follows (Li et al., 2023). Disfluencies are captured by the &- prefix (filled pauses and filler words, e.g., &-um, &-uh), reflecting lexical-access difficulty and hesitation phenomena, and the &+ prefix (phonological fragments, false starts, and stutters), reflecting failures of phonological encoding prior to word retrieval. Extended pauses were captured by CHAT pause markers with three or more dots in parentheses [(...)], which is used in the Pitt corpus as a proxy for silent intervals exceeding approximately 1.5 s. Shorter pause markers [(.) and (..)] were excluded, as they are extended rather than brief hesitations that are clinically associated with processing slowing in AD (Khodabakhsh et al., 2015; Yuan et al., 2020b). Unintelligible segments were captured by xxx (phonetically unclear speech), yyy (speech with recoverable intonation but unclear lexical content), and www (words intentionally not transcribed by the annotator). These distinctions reflect separate neurocognitive substrates. Filled pauses and disfluencies are associated with frontal-executive word-retrieval networks. Extended pauses reflect slowed lexical access linked to hippocampal atrophy and semantic degradation (Khodabakhsh et al., 2015). Unintelligible words reflect degraded phonological and semantic representations at later disease stages.

### 2.4 Dataset construction

Five pre-processed datasets were generated, one per experimental condition. Each condition retains a different subset of CHAT biomarker annotations; the mapping is summarised in Table 3. Each dataset contained 243 AD and 243 control conversations.

### 2.5 Dataset segmentation and split

We segmented each pre-processed dataset using a token-budget windowing strategy to fit within the 512-token context window of BERT-based models. Utterances were greedily packed up to a maximum of 490 tokens, leaving headroom for special BERT tokens such as [CLS] and [SEP]. Chunk boundaries were aligned to conversation turn boundaries rather than fixed turn counts. Trailing overflow chunks with fewer than 100 tokens were discarded to avoid inflating the dataset with near-empty items; approximately seven conversations were affected by this threshold. Three conversations whose second chunks contained 100–275 tokens and 8–15 utterances were retained as genuine items.

Each chunk is a contiguous window of a single participant's utterances from the Cookie-Theft task, not a dialogue act or conversational turn. Only PAR (participant) utterances were retained, so chunks did not interleave INV (investigator) speech. Chunk boundaries were determined by BERT's context limit, not discourse structure. Most participants' responses fit within a single window—only ten conversations produced a second chunk—so the effective unit of analysis remains the participant's full response. Prior work using the Pitt corpus, including Luz et al. (2018) and Liu et al. (2023), similarly treated the full Cookie-Theft response as the unit of analysis, making chunk-level F1 directly comparable.

Each segmented dataset was then partitioned using five-fold stratified cross-validation, with the source file stem as the grouping key. This guaranteed that no participant appears in both the training and test sets within any fold, and that class balance was preserved across folds. Each fold yielded an 80/20 train/test split across participants. From the 80% training portion of each fold, a further group-stratified 10% was held out as an internal validation set used exclusively for early-stopping signal during fine-tuning. This internal validation set was never used for final evaluation.

### 2.6 Model selection and fine-tuning

Three pretrained transformer LMs were compared: A Lite BERT (ALBERT) (Lan et al., 2020), BERT (Devlin et al., 2019), and DistilBERT (Sanh et al., 2019). AlBERT and DistilBERT are BERT-adjacent models derived from the original BERT. AlBERT is a smaller and more memory-efficient model that has fewer parameters compared to BERT with minimal loss in performance (Lan et al., 2020). DistilBERT uses a knowledge distillation method to produce a student model 40% smaller than BERT but retaining 97% of BERT's language comprehension abilities (Sanh et al., 2019). These models were chosen because previous studies have consistently demonstrated their strength and applicability in health-related classification tasks and because of their reduced computational requirements compared to LLM such as Llama (Dubey et al., 2024) and Gemma (Kamath et al., 2025). The three LMs were retrieved from HuggingFace (Wolf et al., 2020). We used all default model weights and configurations as stated in each LM's respective HuggingFace model card. All three models had a maximum context length of 512 tokens. Model architecture details are summarised in Table 4.

**Table 4:** Model configurations.

Model	HuggingFace URL	Params	Layers	Hidden size
ALBERT-base-v2	https://huggingface.co/albert/albert-base-v2	11M	12	768
BERT-base-uncased	https://huggingface.co/google-bert/bert-base-uncased	110M	12	768
DistilBERT-base-uncased	https://huggingface.co/distilbert/distilbert-base-uncased	67M	6	768

Architecture details for the three BERT-based language models.

Each model was fine-tuned to perform the text classification task across all five pre-processed datasets and all five-fold, resulting in a total of 75 fine-tuning runs (3 models × 5 conditions × five-fold). The training parameters are shown in Table 5. Fine-tuning was conducted for up to 20 epochs with early stopping (patience = 3 epochs on internal validation F1). The HuggingFace Trainer was used with a cosine learning-rate schedule and linear warmup. The best checkpoint per run with the highest internal validation F1 was restored before evaluation. ROC curves across all folds and per-model performance metrics heatmaps are reported in Supplementary Figures S1, S2a–c.

**Table 5:** Training parameters for fine-tuning LMs for AD detection.

Parameter	Value
Batch size	4
Epochs (max)	20
Learning rate	2e-05
Optimiser	AdamW
Random seed	4,314
Max sequence length	512 tokens
Early stopping	Patience = 3 (macro-F1)
Class weight modification	None
Compute resource	CUDA/MPS/CPU (auto-detected)
Framework	HuggingFace transformers (Wolf et al., 2020)
Evaluation library	scikit-learn (Pedregosa et al., 2011)

Hyperparameters used during fine-tuning, per model and condition.

### 2.7 Model evaluation

We evaluated each fine-tuned LM on its held-out test set using four standard metrics: accuracy (the proportion of correctly classified instances), precision (the fraction of predicted positive cases that were truly positive), recall (the fraction of true positive cases correctly identified), and macro-averaged F1 score (the harmonic mean of precision and recall, weighted equally across both classes).

The F1 score was selected as the key performance metric to ensure both false positives and false negatives were weighted equally, providing a robust measure of performance for this critical diagnostic task.

Two complementary analyses were conducted. First, to assess whether speech biomarkers improve AD detection, the performance of models trained on each biomarker condition was compared against the No-biomarker baseline, with all five conditions examined (No-biomarker baseline, Disfluencies, Pauses, Unintelligible Words, and All-biomarkers). Second, to identify the best-performing model architecture, the average F1 score of each LM was compared across all five conditions.

### 2.8 Statistical analysis

We tested for significant differences in macro-F1 across biomarker conditions using per-fold scores pooled across all three models (3 models × five-fold = 15 observations per condition). Shapiro–Wilk tests first assessed normality for each condition. We then applied repeated-measures ANOVA across the five biomarker conditions, with Greenhouse-Geisser correction if Mauchly's test rejected sphericity. Directional Wilcoxon signed-rank *post-hoc* tests compared each condition against pauses (reference) at α = 0.05 (uncorrected). All analyses were conducted in Python using the SciPy and Pingouin libraries.

## 3 Results

### 3.1 Effect of speech biomarkers on AD detection

**Figure 2:**
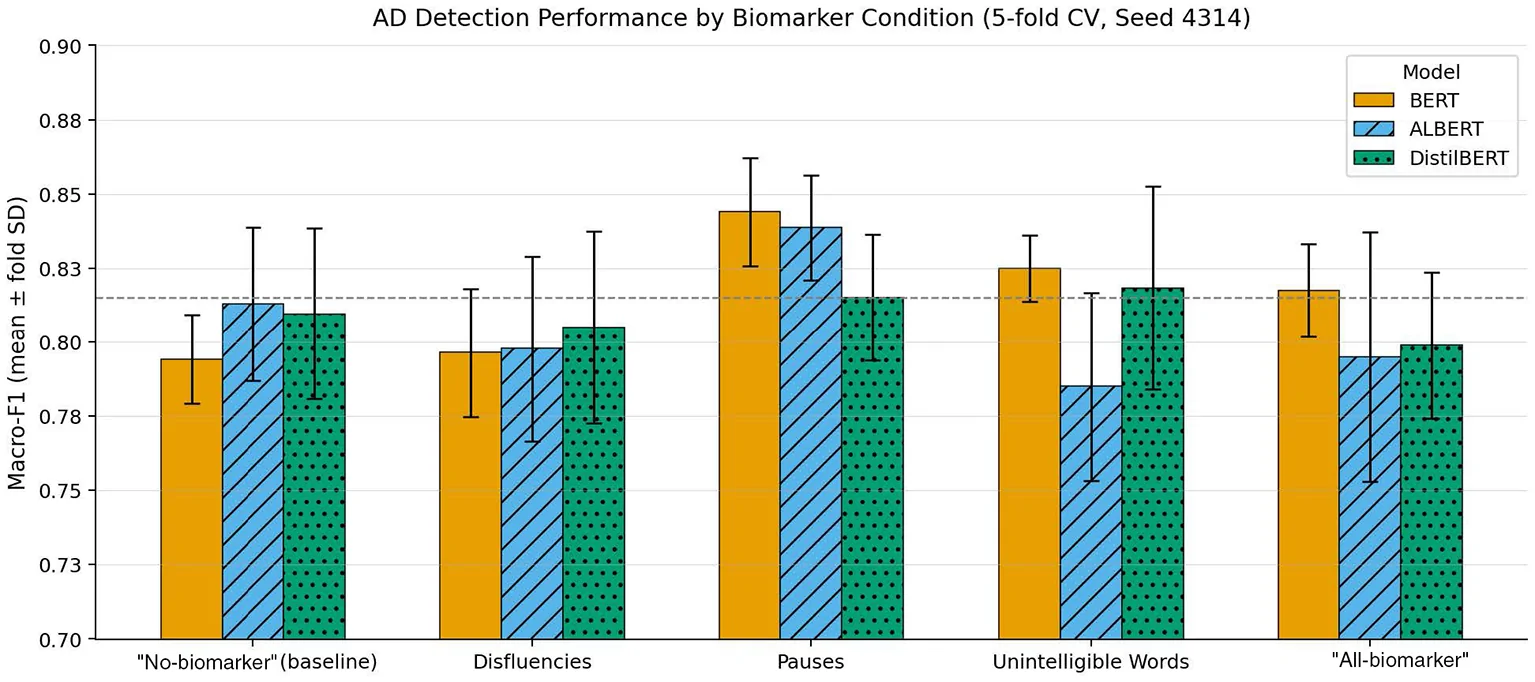
Macro-F1 by biomarker condition and model architecture (five-fold CV). Mean macro-F1 ± within-model fold standard deviation per model across five biomarker conditions. The dashed horizontal line marks the cross-model average for the No-biomarkers baseline (0.8056). Pauses consistently yielded the highest F1 per model; disfluencies and All-biomarker conditions fall at or below baseline.

**Figure 3:**
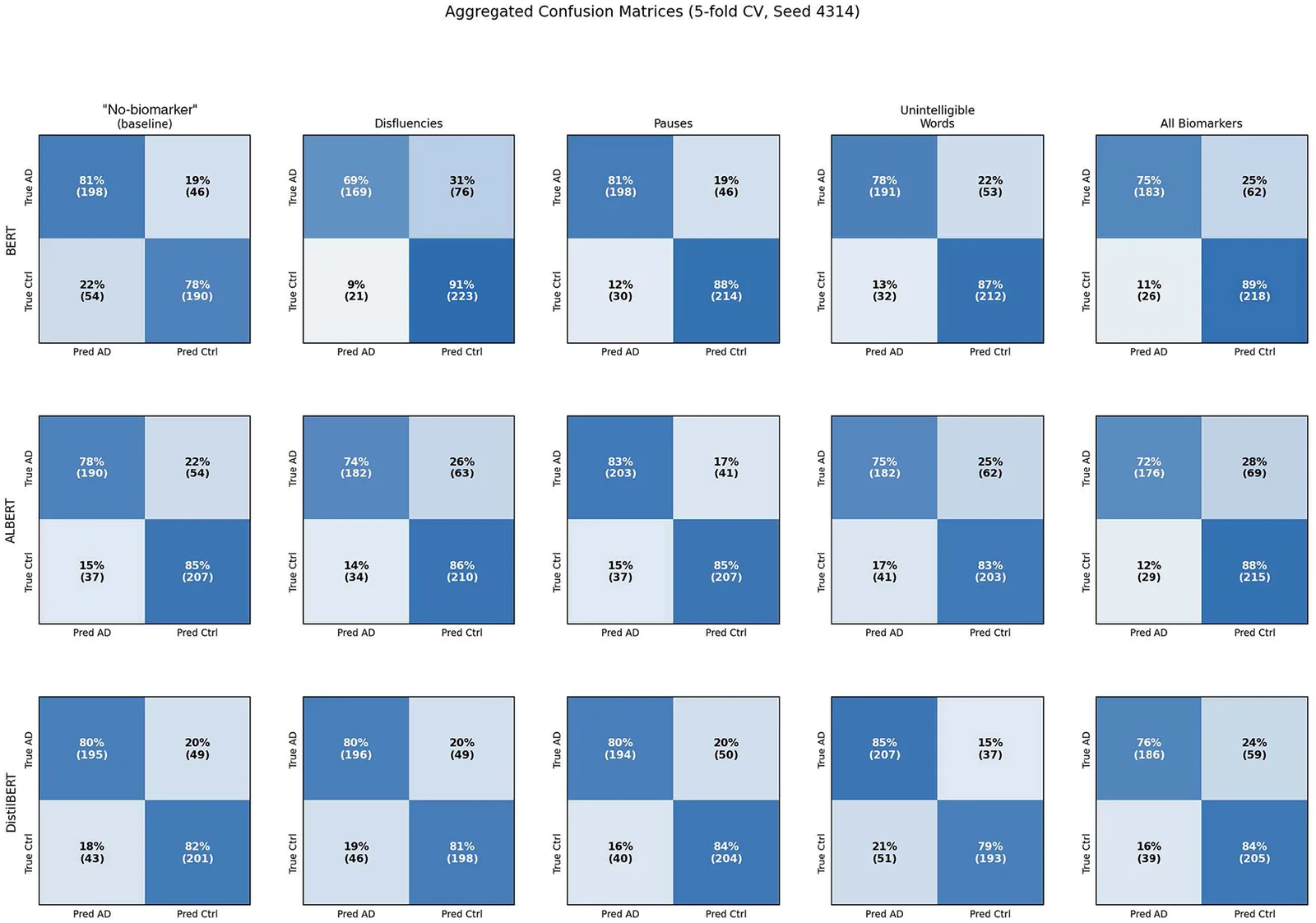
Aggregated confusion matrices for the AD detection task (five-fold CV). Confusion matrices per model (BERT, AlBERT, DistilBERT) and condition (No-biomarkers, Disfluencies, Pauses, Unintelligible Words, All-biomarkers). Cell values show row-normalised percentages and absolute counts. True positives indicate correct AD detection; true negatives indicate correct control classification.

Speech biomarkers improved LM performance in AD detection relative to the No-biomarkers baseline (F1 = 0.8056; Table 6). Pauses yielded the strongest signal (F1 = 0.8326). Figure 2 shows the cross-condition comparison across all three models; confusion matrices are shown in Figure 3.

**Table 6:** Mean macro-F1 and mean AUC-ROC of fine-tuned LMs on the AD detection task (five-fold CV).

Dataset	AlBERT	BERT	DistilBERT	Avg. F1	Avg. AUC-ROC
No-biomarker (baseline)	**0.8129**	0.7942	0.8097	0.8056	0.9080
Disfluencies	0.7978	0.7964	**0.8050**	0.7997	0.9008
Pauses	0.8387	**0.8440**	0.8151	**0.8326**	**0.9145**
Unintelligible words	0.7851	**0.8250**	0.8183	0.8094	0.9040
All-biomarker	0.7950	**0.8175**	0.7989	0.8038	0.9002
**Avg. F1 per model**	0.8059	**0.8154**	0.8094		
**Avg. AUC per model**	0.9011	**0.9141**	0.9013		

Per-condition and per-architecture results across five-fold. Bold values indicate best performance per column.

### 3.2 Model architecture comparison

BERT lead all settings (mean F1 = 0.8154), followed by DistilBERT (mean F1 = 0.8094), and ALBERT (mean F1 = 0.8059). Its strongest result was the Pauses condition [F1 = 0.8440, 95% CI: (0.8280, 0.8600), AUC-ROC = 0.9196]. All three models remained within 0.010 F1 of each other on average. Full per-model metrics—AUC-ROC, 95% confidence intervals, sensitivity, and specificity—appear in Supplementary Table S3.

### 3.3 Statistical significance

Shapiro-Wilk tests did not reject normality for any condition (all *p*>0.05). Repeated-measures ANOVA revealed a significant effect of biomarker condition on macro-F1 [*F*_(4, 56)_ = 2.560, *p* = 0.048, η^2^ = 0.142; Mauchly's test confirmed sphericity, no correction applied]. Wilcoxon signed-rank *post-hoc* tests confirmed that the Pauses condition significantly outperformed all other conditions: Disfluences (*W* = 102.0, *p* = 0.008), Unintelligible Words (*W* = 96.0, *p* = 0.021), All-biomarkers (*W* = 96.0, *p* = 0.021), and the No-biomarkers baseline (*W* = 104.5, *p* = 0.006).

### 3.4 Robustness analysis

*Post-hoc* subgroup analysis of model predictions revealed apparent differences in classification F1 between probable AD (mean F1 = 0.87 across models and conditions) and possible AD (mean F1 = 0.93; Supplementary Table S2). The possible AD subgroup comprised only 21 participants (approximately four per test fold), however, making these estimates unreliable and precluding firm subgroup-specific conclusions.

To assess robustness to diagnostic criterion, we ran a sensitivity analysis restricting the positive class to probable AD participants only (*n* = 222; Supplementary Table S5). We used DistilBERT on the Pauses condition—the best-performing biomarker—and the probable-only model achieved F1 = 0.8027 (95% CI: [0.7835, 0.8219], AUC-ROC = 0.8996), compared to F1 = 0.8151 (AUC-ROC = 0.9081) when the full ProbableAD + PossibleAD positive class was used. The modest reduction (ΔF1 = −0.012) confirmed that the primary classification signal derives from probable AD cases.

## 4 Discussion

### 4.1 Principal results

Biomarker type drived performance more than architecture. Pauses yielded the strongest signal; Disfluencies degraded it. All three models remained within 0.010 F1 of each other on average.

Pauses (F1 = 0.8326) emerged as the dominant biomarker, consistent with prior work (Khodabakhsh et al., 2015; Liu et al., 2023; Yuan et al., 2020b) establishing extended pauses as a well-characterised indicator of AD. This aligns with neurological evidence that pauses reflect word-finding difficulties and reduced processing speed, which are early hallmarks of AD-related cognitive decline (Khodabakhsh et al., 2015). This likely explains why this feature yields the clearest discriminatory signal for BERT-based LMs.

We also examined raw biomarker frequency differences between AD and control groups using Mann–Whitney *U* tests. Unintelligible words were significantly more frequent in the AD group (mean 0.383 vs. 0.169 tokens per transcript; *U* = 33589, *p* < 0.001, *r* = −0.138), while disfluency (*p* = 0.248) and long-pause (*p* = 0.569) counts did not differ significantly. This is a notable dissociation: pauses yield the strongest classification signal (F1 = 0.8326) despite showing no significant raw frequency difference at the transcript level. The models are not simply counting pauses: they appear to be capturing where and how pauses fall relative to surrounding speech. This contextual sensitivity likely explains why LMs outperform frequency-based approaches on this task.

Combining all three biomarkers degraded performance (F1 = 0.8038) relative to pauses alone (F1 = 0.8326). Not all features contributed, introduced, confounded the classification signal. The effect was not uniform across architectures, however: BERT's All-biomarkers condition (F1 = 0.8175) exceeded its No-biomarkers baseline (F1 = 0.7942), whereas ALBERT (F1 = 0.7950 vs. 0.8129) and DistilBERT (F1 = 0.7989 vs. 0.8097) both declined. BERT's higher capacity may allow it to integrate heterogeneous biomarker signals that smaller models cannot as effectively exploit.

Liu et al. (2023) reported F1 up to 0.89 using acoustic and textual features; Luz et al. (2018) achieved 0.75–0.80 using conversational speech features. Our Pause-only results (F1 = 0.8326) fall within this range.

The ADReSS 2020 shared task (Luz et al., 2018) provides a more direct comparison: 108 participants from the same Pitt corpus, a challenge baseline of F1 = 0.63, and a best-system result of F1 = 0.815 (AUC = 0.89) using both acoustic and text features. Text-only BERT approaches on similar subsets have reached F1 = 0.75–0.84. Our Pause-only model (F1 = 0.8326) matches or exceeds that range using a single sparse biomarker. Targeted biomarker isolation captures most of the discriminative signal, accounting for differences in dataset splits and balancing across studies.

Of the three biomarkers, pauses provide the strongest discriminative signal, unintelligible words add modest value, and disfluencies do not contribute above the baseline. Per-class error analysis reveals why: for disfluencies, BERT achieves high specificity (0.9139) but low sensitivity (0.6898), meaning it rarely misclassifies controls as AD but misses a substantial proportion of true AD cases. This is a clinically unfavourable trade-off relative to pauses (sensitivity 0.8115, specificity 0.8770), which achieves a more balanced error profile (Supplementary Table S3).

Pause dominance also appears to generalise beyond AD. Corcoran et al. (2020) found that pauses were the most effective biomarker for both schizophrenia and AD classification. This suggests that pause features may generalise across speech-impaired conditions.

### 4.2 Model performance comparison

BERT's superior average performance (mean F1 = 0.8154) reflects its balance of model capacity and regularisation. BERT Achieved the highest Pauses result (F1 = 0.8440) and the strongest All-biomarkers result (F1 = 0.8175), indicating its ability to extract signal from richer annotation contexts. DistilBERT's comparable performance (mean F1 = 0.8094) with 40% fewer parameters demonstrates that distillation successfully preserves discriminative capacity for this task. ALBERT trails slightly (mean F1 = 0.8059). But all three models remain within 0.010 F1 of each other on average. Biomarker quality, not architecture, is the dominant factor driving performance.

A pause-only model achieving F1 = 0.8326 requires nothing more than a brief recorded speech sample to flag individuals for further clinical evaluation. This makes it viable for telehealth platforms and general-practice consultations, where access to specialist neuropsychological assessment is limited. But deployment requires more than a lightweight input format. BERT's consistent performance across all biomarker conditions shows the architecture is not brittle to annotation choice. Yet, clinical application still requires validation on larger, more diverse datasets and prospective cohorts before deployment.

### 4.3 Limitations

Each per-fold test set is small (approximately 50 chunks under five-fold cross-validation), producing within-fold F1 standard deviations of up to 0.04. Biomarker rankings are consistent across all three models, and the statistical tests in Section 3.3 confirm their significance. But external validation on larger, independent cohorts is essential before generalising these conclusions.

The optimal token-budget windowing strategy (chunk size, boundaries) for this task remains unexplored and should be investigated. Token-based chunking enables efficient processing, but assumes that AD-relevant information is contained within those local chunks. Previous literature on dialogue analysis has indicated that cognitive decline may also be reflected in wider windows of texts, including conversational organisation and repair sequences. The present representation may under-use discourse-level information, representing an avenue for future research.

Only three speech biomarkers were examined. Future work should incorporate more varied features across modalities, including vocal prosody beyond pauses (e.g., pitch, rate, energy).

External validation on independent datasets (e.g., ADReSS, other DementiaBank subsets) is required before strong conclusions can be drawn. All analyses relied exclusively on the Pitt subset of DementiaBank; results may differ with other corpora. Subgroup estimates and robustness checks are reported in Section 3.4; the small possible AD subgroup (*n* = 21, approximately four participants per test fold) is a data-level constraint that limits subgroup-specific interpretation.

The AD and control groups in the Pitt corpus differ in mean age (AD: 72.0 ± 8.3 years; controls: 64.9 ± 7.7 years), a gap of approximately seven years. Extended pauses reflect neurodegeneration rather than normal ageing; long pause ratios are near-zero in healthy older adults (Khodabakhsh et al., 2015). But the age gap introduces a potential confound: some inter-group speech differences may partly reflect age-related rather than disease-specific processes. Future work should investigate designs with stricter age matching.

Individual MMSE scores were not available in the transcript-level metadata files; cohort-level distributions are reported in the DementiaBank characterisation study (Lanzi et al., 2023). Future work should correlate per-participant predicted AD probability with MMSE score. Prior work in the ADReSS shared task explored MMSE regression from transcript features, achieving *R*^2^ values of 0.30–0.50. This indicates that speech captures a meaningful but incomplete portion of cognitive test performance. Such an analysis would require access to the consortium-level metadata.

The study was restricted to English-speaking patients, limiting generalisability to the broader global AD population.

### 4.4 Recommendations and future work

Non-English AD research remains scarce. Most studies continue to rely on DementiaBank. The broader field is shifting towards ensemble transformers, LLMs, and multimodal systems for neurodegenerative disease detection. Our choice of BERT-scale models was deliberate: BERT, AlBERT, and DistilBERT achieve strong performance (F1 = 0.81–0.82) with a fraction of the compute required by LLMs—a critical property for clinical deployment in resource-constrained settings. For focused biomarker-driven tasks where these models already approach performance saturation, the marginal gains from LLMs do not justify their overhead. However, future work should probe whether LLMs surface different biomarker orderings or signals that BERT-scale models miss.

Our methodology targets breaks in regular speech patterns rather than language-specific vocabulary, making it directly applicable to non-English cohorts. The pipeline is not tied to AD-specific transcripts; it generalises to other forms of cognitive decline, including schizophrenia and depression. LMs can then be adapted to these contexts through prompting, retrieval-augmented generation, fine-tuning, or pre-training (Zhou S. et al., 2025).

### 4.5 Conclusions

Systematic biomarker isolation improves AD detection using LMs. Comparing individual vs. combined biomarkers reveals a clear pattern: pauses are robustly discriminatory (F1 = 0.8326), but combining features degrades performance on average (F1 = 0.8038 vs. 0.8326). BERT is the exception—its All-biomarkers condition exceeds its own No-biomarker baseline. BERT showed the strongest performance overall (mean F1 = 0.8154), followed by DistilBERT (F1 = 0.8094) and ALBERT (F1 = 0.8059). Targeted biomarker selection, not feature consolidation, optimises both performance and interpretability. The methodology is not language-specific or disease-specific. External validation on larger, more diverse cohorts is essential before clinical application. LMs adapted to verbal patterns alone detect early AD with clinically competitive accuracy. A pause-focused model is scalable, low-cost, and integrable into ageing services to support earlier referrals, reduce diagnostic delays, and personalise care.

## Data Availability

Publicly available datasets were analyzed in this study. This data can be found at: the DementiaBank Pitt corpus is publicly available through TalkBank but requires researcher registration and the acceptance of a data use agreement prior to access. Link: https://talkbank.org/dementia/access/Repository. TalkBank/DementiaBank Accession: Pitt Corpus (DementiaBank) https://doi.org/10.21415/CQCW-1F92.
