# Supplementary material for: Utilising speech-derived biomarkers to detect Alzheimer's disease with BERT-based language models: a machine learning study
Source: Front Artif Intell. 2026 Aug 31;9:1875702. doi: 10.3389/frai.2026.1875702 (PMC13572675; doi:10.3389/frai.2026.1875702)
Supplement: Supplementary file 1 [file Presentation_1.pdf]

Supplementary Material

STUDY OVERVIEW

This supplementary material presents ROC curves across all 5 cross-validation folds, per-model performance metrics heatmaps, and statistical significance test results for all three language models (BERT, AlBERT, DistilBERT) across all five biomarker conditions. All results in the main manuscript derive from 5-fold stratified cross-validation on participant-only speech transcripts: 243 probable/possible AD patients and 243 matched controls from the DementiaBank Pitt corpus.

APPENDIX A: STATISTICAL SIGNIFICANCE TESTING

We tested whether F1 differences across biomarker conditions were statistically significant using per-fold scores from all three models and all five folds (15 paired observations per condition).

Normality and Statistical Tests

All five conditions satisfied the normality assumption (Shapiro-Wilk; all $p > 0.05$), supporting parametric testing. We ran a Repeated Measures ANOVA to test whether mean F1 differed across the five biomarker conditions.

Results

The Repeated Measures ANOVA detected a significant effect of condition: $F(4, 56) = 2.560$, $p = 0.048$, $\eta^2_{\text{adj}} = 0.142$. We followed up with one-sided Wilcoxon signed-rank tests ($\alpha = 0.05$) to evaluate directional hypotheses (Table S1).

Pauses significantly outperformed every other condition: the no-biomarkers baseline ($p = 0.0057$, ΔF1 $= +0.0270$), disfluencies ($p = 0.0075$, ΔF1 $= +0.0328$), unintelligible words ($p = 0.0206$, ΔF1 $= +0.0231$), and all biomarkers ($p = 0.0206$, ΔF1 $= +0.0288$). Effect sizes were small-to-medium (rank-biserial $r = 0.129$–0.200).

Table S1. Wilcoxon signed-rank post-hoc tests: pauses vs. other conditions. One-sided directional tests (pauses > other), $\alpha = 0.05$. Δmean = difference in mean F1; r (RBC) = rank-biserial correlation effect size.

Comparison	W	p-value	Δmean	r (RBC)	Significant?
Pauses > No Biomarkers	104.5	0.0057	+0.0270	0.129	Yes
Pauses > Disfluencies	102	0.0075	+0.0328	0.150	Yes
Pauses > Unintelligible Words	96	0.0206	+0.0231	0.200	Yes
Pauses > All Biomarkers	96	0.0206	+0.0288	0.200	Yes

Table S2. Post-hoc subgroup F1: probable AD vs. possible AD. Macro-F1 computed separately for probable AD ($n = 222$–224 across folds) and possible AD ($n = 21$ total, approximately four per test fold) participants, with values aggregated across all five cross-validation folds per model–condition combination. The possible AD subgroup is far too small for reliable estimates; we report these values for transparency in response to reviewer queries and they do not constitute evidence of genuine subgroup-specific performance differences.

Model	Condition	Probable AD F1	Possible AD F1
ALBERT	All Biomarkers	0.8364	0.8333
	Disfluencies	0.8454	0.9231
	No Biomarkers	0.8680	0.9500
	Pauses	0.9015	0.9756
	Unintelligible	0.8446	0.9500
BERT	All Biomarkers	0.8483	0.9231
	Disfluencies	0.8148	0.8333
	No Biomarkers	0.8933	0.9231
	Pauses	0.8933	0.9231
	Unintelligible	0.8737	0.9231
DistilBERT	All Biomarkers	0.8542	0.9500
	Disfluencies	0.8828	0.9500
	No Biomarkers	0.8794	0.9756
	Pauses	0.8822	0.9231
	Unintelligible	0.9122	0.9756

Table S3. Comprehensive per-model performance metrics (5-fold CV, ad_only dataset). Mean macro-F1 ($\pm$ fold SD), 95% confidence interval (CI; $\pm 1.96 \times \mathrm{SD}/\sqrt{5}$), mean AUC-ROC, sensitivity (AD recall), specificity (control recall), AD precision, and control precision for each model–condition combination. We pool sensitivity and specificity across folds from total TP, FP, FN, TN counts. Bold values indicate the highest mean F1 per condition. Figures S2a–S2c show full per-fold AUC-ROC, accuracy, and MCC heatmaps.

Condition	LM	F1 ± SD	95% CI	AUC-ROC	Sens.	Spec.	Prec. (AD / Ctrl)
No Biomarkers	ALBERT	**0.8129** ± 0.0259	±0.0227	0.9113	0.7787	0.8484	0.8370 / 0.7931
	BERT	0.7942 ± 0.0150	±0.0131	0.8993	0.8115	0.7787	0.7857 / 0.8051
	DistilBERT	0.8097 ± 0.0288	±0.0253	0.9135	0.7992	0.8238	0.8193 / 0.8040
Disfluencies	ALBERT	0.7978 ± 0.0312	±0.0273	0.8890	0.7429	0.8607	0.8426 / 0.7692
	BERT	0.7964 ± 0.0216	±0.0189	0.9225	0.6898	0.9139	0.8895 / 0.7458
	DistilBERT	**0.8050** ± 0.0324	±0.0284	0.8909	0.8000	0.8115	0.8099 / 0.8016
Pauses	ALBERT	0.8387 ± 0.0177	±0.0155	0.9157	0.8320	0.8484	0.8458 / 0.8347
	BERT	**0.8440** ± 0.0182	±0.0160	0.9196	0.8115	0.8770	0.8684 / 0.8231
	DistilBERT	0.8151 ± 0.0213	±0.0187	0.9081	0.7951	0.8361	0.8291 / 0.8031
Unintelligible Words	ALBERT	0.7851 ± 0.0317	±0.0278	0.8863	0.7459	0.8320	0.8161 / 0.7660
	BERT	**0.8250** ± 0.0112	±0.0098	0.9104	0.7828	0.8689	0.8565 / 0.8000
	DistilBERT	0.8183 ± 0.0342	±0.0300	0.9153	0.8484	0.7910	0.8023 / 0.8391
All Biomarkers	ALBERT	0.7950 ± 0.0421	±0.0369	0.9033	0.7184	0.8811	0.8585 / 0.7570
	BERT	**0.8175** ± 0.0157	±0.0137	0.9187	0.7469	0.8934	0.8756 / 0.7786
	DistilBERT	0.7989 ± 0.0246	±0.0216	0.8786	0.7592	0.8402	0.8267 / 0.7765

Sensitivity = AD recall (TP rate); Specificity = control recall (TN rate). Sensitivity and specificity pooled across all five folds from total TP, FP, FN, TN counts.

Table S4. Mann-Whitney U test results comparing raw biomarker token frequency between AD ($n = 243$) and control ($n = 243$) transcripts. We derived counts from the `all_biomarkers` preprocessed JSONL, which retains all three biomarker types. Effect size r is the rank-biserial correlation ($r = 1 - 2U/(n_1 n_2)$).

Biomarker	AD mean	AD med	Ctrl mean	Ctrl med	U	p	r	Sig.
Disfluencies	5.066	3	4.058	3	31300.5	0.248	-0.060	No
Long pauses ($\geq$3)	0.099	0	0.070	0	29909.5	0.569	-0.013	No
Unintelligible words	0.383	0	0.169	0	33589.0	$<$0.001	-0.138	Yes

Long pauses defined as CHAT markers with 3 or more dots (proxy for pauses > 1.5 s). Significance threshold $\alpha = 0.05$ (two-sided).

Table S5. Sensitivity analysis: probable AD vs controls (DistilBERT, pauses condition, 5-fold CV). Performance when restricting the positive class to probable AD participants only ($n = 222$ AD, $n = 243$ controls; see main text §4.3). Compare to DistilBERT ad_only pauses (F1 = 0.8151, AUC = 0.9081) which includes both probable and possible AD.

Fold	F1	AUC-ROC
0	0.8122	0.9327
1	0.8333	0.9032
2	0.8095	0.9119
3	0.7902	0.8906
4	0.7684	0.8599
**Mean $\pm$ SD**	**0.8027 $\pm$ 0.0219**	**0.8996 $\pm$ 0.0241**
**95% CI**	$\pm$0.0192	$\pm$0.0211

95% CI computed as $\pm 1.96 \times \text{SD}/\sqrt{5}$. Note: the probable_only dataset has 222 AD files and 243 controls; since $n_{\text{AD}} < n_{\text{ctrl}}$, all AD files are used (no downsampling) and all 243 controls are used, yielding a slightly unbalanced dataset (222 AD vs 243 control). Macro-F1 accounts for this imbalance.

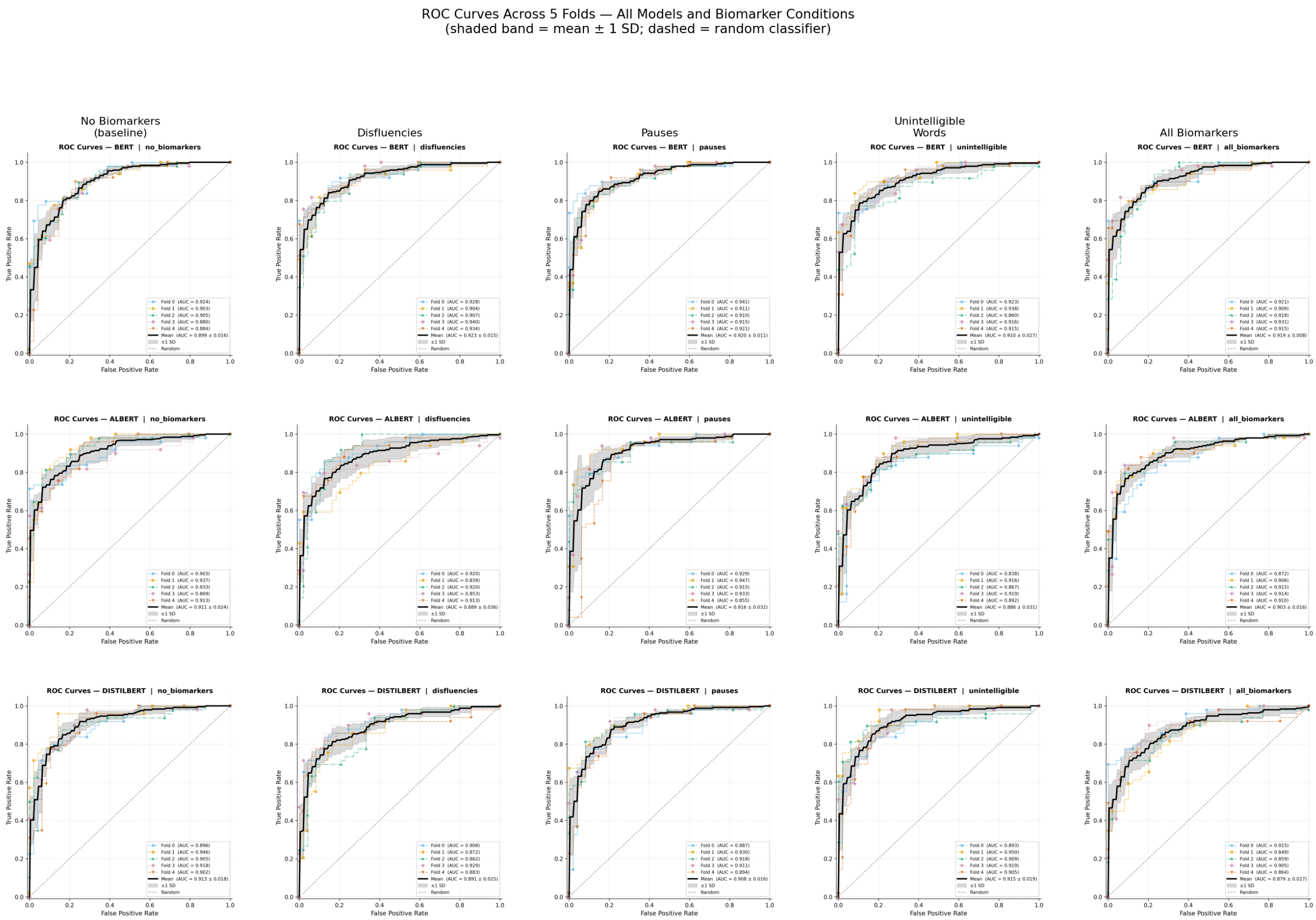

Figure S3. ROC curves across all 5 cross-validation folds for all models and biomarker conditions. Rows correspond to model architecture (BERT, ALBERT, DistilBERT); columns correspond to biomarker condition (No Biomarkers, Disfluencies, Pauses, Unintelligible Words, All Biomarkers). Each panel shows the ROC curves for the five individual folds (thin lines) together with the mean ROC curve $\pm$ 1 SD shading. Each panel labels the mean AUC. Dashed diagonal: random classifier.

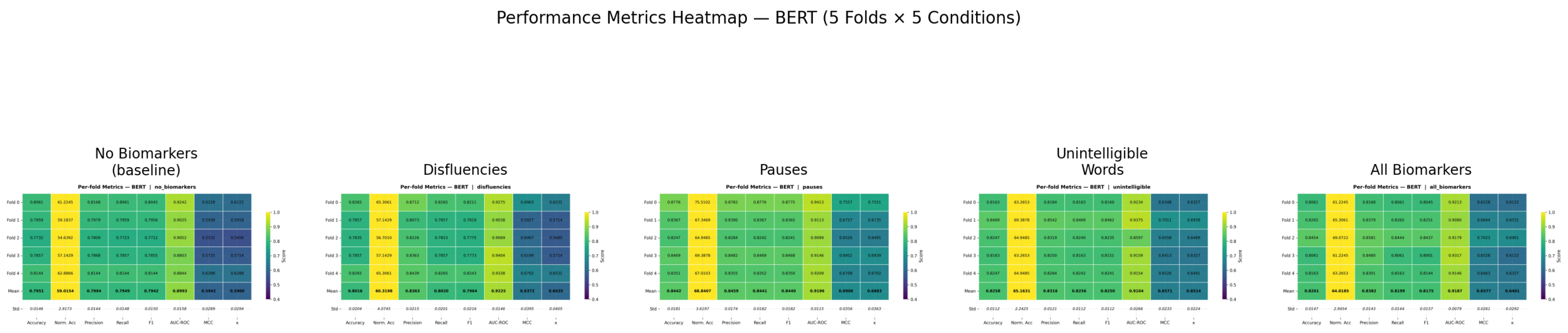

Figure S4. Performance metrics heatmap — BERT (5 folds × 5 conditions). Each panel shows macro-F1, AUC-ROC, accuracy, and MCC across all five folds for one biomarker condition (No Biomarkers, Disfluencies, Pauses, Unintelligible Words, All Biomarkers), enabling direct assessment of fold-to-fold variability.

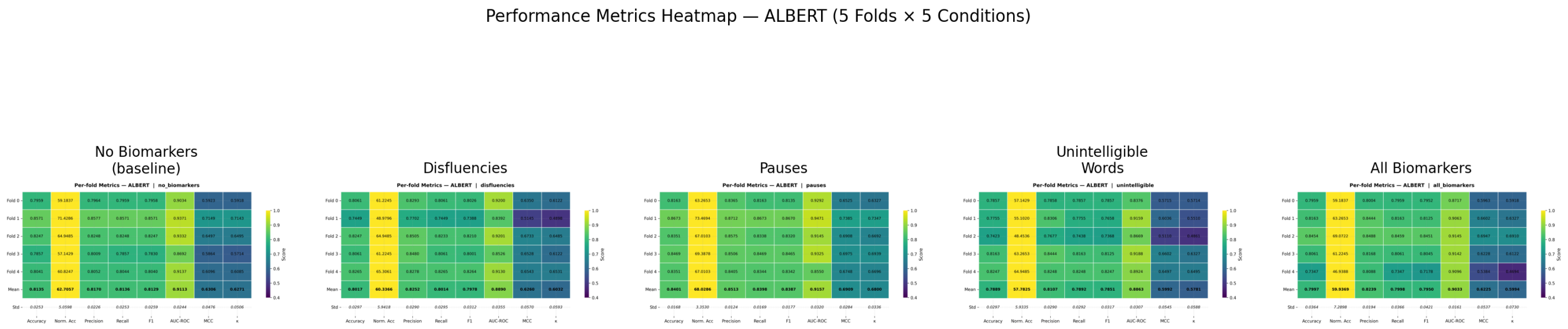

Figure S5. Performance metrics heatmap — ALBERT (5 folds × 5 conditions). Layout identical to Figure S2a; rows index metric, columns index fold.

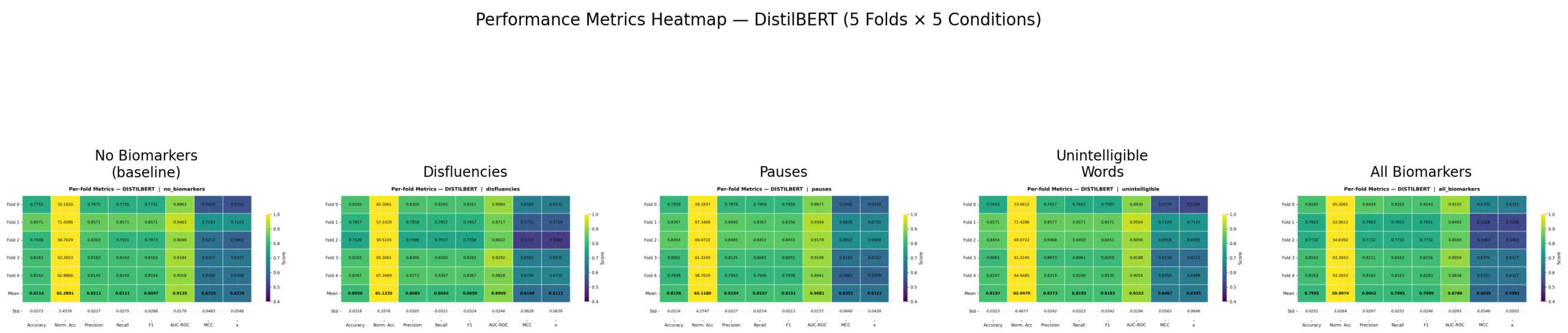

Figure S6. Performance metrics heatmap — DistilBERT (5 folds × 5 conditions). Layout identical to Figure S2a; rows index metric, columns index fold.
